# Care during the third stage of labour: obstetricians views and practice in an Albanian maternity hospital

**DOI:** 10.1186/1471-2393-10-4

**Published:** 2010-01-26

**Authors:** Astrit Bimbashi, Eriseida Ndoni, Anika Dokle, Lelia Duley

**Affiliations:** 1 University Hospital of Obstetrics and Gynecology 'Koço Gliozheni', Tirana Albania; 2 Centre for Epidemiology and Biostatistics, University of Leeds, UK

## Abstract

**Background:**

Relatively little is known about current practice during the third stage of labour in low and middle income countries. We conducted a survey of attitudes and an audit of practice in a large maternity hospital in Albania.

**Methods:**

Survey of 35 obstetricians and audit of practice during the third stage was conducted in July 2008 at a tertiary referral hospital in Tirana. The survey questionnaire was self completed. Responses were anonymous. For the audit, information collected included time of administration of the uterotonic drug, gestation at birth, position of the baby before cord clamping, cord traction, and need for resuscitation.

**Results:**

77% (27/35) of obstetricians completed the questionnaire, of whom 78% (21/27) reported always or usually using active management, and 22% (6/27) always or usually using physiological care. When using active management: 56% (15/27) gave the uterotonic after cord clamping; intravenous oxytocin was almost always the drug used; and 71% (19/27) clamped the cord within one minute. For physiological care: 42% (8/19) clamped the cord within 20 seconds, and 96% (18/19) within one minute. 93% would randomise women to a trial of early versus late cord clamping.

Practice was observed for 156 consecutive births, of which 26% (42/156) were by caesarean section. A prophylactic uterotonic was used for 87% (137/156): this was given after cord clamping for 55% (75/137), although timing of administration was not recorded for 21% (29/137). For 85% of births (132/156) cord clamping was within 20 seconds, and for all babies it was within 50 seconds. Controlled cord traction was used for 49% (76/156) of births.

**Conclusions:**

Most obstetricians reported always or usually using active management for the third stage of labour. For timing and choice of the uterotonic drug, reported practice was similar to actual practice. Although some obstetricians reported they waited longer than one minute before clamping the cord, this was not observed in practice. Controlled cord traction was used for half the births.

## Background

Traditionally active management was defined as administration of a prophylactic uterotonic drug, immediate clamping of the umbilical cord, and controlled cord traction [1,2]. Immediate cord clamping restricts placental transfusion, which is the flow of blood from the placenta and cord to the baby at birth. There is now evidence that this may have harmful effects for the child [3,4]. Although the evidence is not conclusive,[5] various international agencies such as the World Health Organisation (WHO), the International Confederation of Midwives (ICM) and the International Federation of Gynecology and Obstetrics (FIGO) now recommend deferring cord clamping for three minutes [6,7].

The main aim of care during the third stage of labour is to prevent postpartum haemorrhage. Active management reduces the relative risk of postpartum haemorrhage by around 60%, compared with physiological care [2]. Much of this reduction in risk is due to the use of a prophylactic uterotonic drug, ideally oxytocin[7,8] although when is best to give it remains uncertain [9]. The impact of other commonly used components of active management, such as controlled cord traction,[10] and uterine massage[11] is also unclear.

Developing effective strategies to improve care during labour requires reliable evidence about the effects of interventions, and an understanding of current practice. Albania is a lower middle income country with a young population, 5% aged 60 years or more compared to 21% in the UK, and high fertility (fertility rate 6.8 compared to 1.6 in the UK) [12]. Maternal mortality is 55 deaths per 100,000 live births [12]. Relatively little is known about current practice during the third stage in low and middle income countries [13]. We conducted a survey of obstetricians' views and observed current practice for care during the third stage of labour at a large maternity hospital in Tirana, Albania.

## Methods

A survey of obstetricians and audit of practice during the third stage was conducted over a two week period in July 2008 at the University Hospital of Obstetrics and Gynecology 'Koço Gliozheni', Tirana. This unit is a tertiary referral centre and teaching hospital with 4,100 births per year. There are around 9,500 births per year in Tirana, and 33,200 in Albania. All births at the University Hospital are attended by an obstetrician. Although normal births are conducted by midwives, an obstetrician is always present. Postgraduate trainees in obstetrics often also attend vaginal and caesarean births as observers. The hospital does not have a guideline for care during the third stage.

All 35 obstetricians working in the hospital during the study period were invited to complete a one page questionnaire asking about their current practice for care during the third stage of labour. The questionnaire was adapted from a similar survey used in the UK[14] and included open ended and closed response questions. Responses were anonymous. Returned, completed questionnaires were considered indicative of consent to participate.

An audit of practice during the third stage was conducted during a 2 week period in July 2008. Two weeks was selected as being feasible to achieve, and sufficient to be representative. Consecutive deliveries were observed by a team of six third year postgraduate trainees, trained by one of us (AB) in the use of the audit form. The trainees conducted the audit during their normal working hours. Normal practice is for these trainees to attend deliveries as an observer, their presence during the conduct of this audit was unlikely to have influenced care during the third stage. Information was recorded on a pre-specified form, adapted from a similar one used in the UK [14]. Data collected included time of administration of the uterotonic, gestation at birth, position of the baby before cord clamping, cord traction, and need for resuscitation. Timing of cord clamping was measured with a stop watch. Data were anonymous and confidential.

Ethics approval was not required, as both studies were considered to be audit. Data were checked for completeness and accuracy.

## Results

Of the 35 obstetricians, 27 (77%) completed the questionnaire. Seventy eight percent of obstetricians reported they always or usually use active management, and 22% that they always or usually use physiological management (Table 1). When using active management 56% of obstetricians gave the uterotonic after cord clamping, and intravenous oxytocin was almost always the drug used. There was variation in when the cord was clamped, although most reported clamping within one minute. When using physiological care, 42% (8/19) clamped the cord within 20 seconds. 93% (27/29) of obstetricians thought there should be trials comparing immediate with deferred cord clamping, all of whom were willing to randomise to such a trial.

**Table 1:** Survey of obstetricians

	n = 27 (%)	
***How often do you use active management of the 3rd stage of labour?***		
always or usually	21	(78)
sometimes	4	(15)
rarely	2	(7)
never	-	
		
***When you use active management***		
*When do you normally give the prophylactic uterotonic?*		
with anterior shoulder	4	(15)
with delivery of the baby	3	(11)
after birth of baby, before cord clamping	5	(19)
after birth of baby, after cord clamping	15	(56)
		
*Which uterotonic do you normally use?*		
oxytocin iv	25	(93)
methergine* im	2	(7)
		
*How long after birth of a term baby do you normally clamp the cord?*		
within 20 seconds	6	(22)
within 20-60 seconds	13	(49)
within 1-3 minutes	7	(26)
after cessation of pulsation	1	(4)
		
***How do you define early cord clamping?***		
within 10 seconds	10	(37)
within 20 seconds	11	(41)
within 30 seconds	4	(15)
within 1 minutes	2	(7)
		
***How do you define late cord clamping?***		
after 30 seconds	1	(4)
after 1 minute	11	(41)
after 3 minutes	10	(37)
after cessation of pulsation	5	(19)
		
**How often do you use physiological management of the third stage?**		
always or usually	6	(22)
sometimes	6	(22)
rarely	9	(33)
never	6	(22)
		
*When you use physiological management, how long after birth of a term baby do you normally clamp the cord?*		
1-20 seconds	8	
21-60 seconds	10	
after one minute	1	
		
***Would be willing to randomise to a trial of early versus late clamping***	25	(93)

Practice was observed for 156 births. Of these, 27% (95% confidence interval 21% to 33%) were by caesarean section and 10% (6% to 14%) were before 37 competed weeks (Table 2). A prophylactic uterotonic was administered for 87% (83% to 93%) of births: for 55% (75/137) this was given after cord clamping, although for a fifth of births (29/137) the timing of administration was not recorded. Cord clamping was usually within 20 seconds, and for all babies was within 50 seconds.

**Table 2:** Observation of practice during the third stage

	Total n = 156 (%)
*Gestation at birth*	
preterm	16 (10)
term	140 (90)
	
*Mode of delivery*	
vaginal	114 (74)
Caesarean section	42 (26)
	
*Uterotonic used during 1^st ^or 2^nd ^stage*	31 (20)
	
*Prophylactic uterotonic used in 3^rd ^stage*	137 (87)
oxytocin iv	101
oxytocin iv and methergine* im	36
	
with anterior shoulder	2
with birth of the baby	10
after birth of the baby, before cord clamping	21
after birth of the baby, after cord clamping	75
not stated	29
	
*Position of the baby when cord clamped^† ^*	
on the woman's abdomen	6 (5)
held by person doing the delivery	109 (95)
	
*Time from birth to cord clamping (seconds)*	
11-20	132 (85)
21-30	19 (12)
31-40	4 (3)
41-50	1 (1)
	
*Controlled cord traction *	76 (49)
	
*Baby required resuscitation at birth*	10 (6)

iv = intravenous; im = intramuscular

preterm = < 37 completed weeks gestation; term = ≥ 37 completed weeks

† for vaginal deliveries only; * methylergonovine maleate, a semi-synthetic ergot alkaloid

Controlled cord traction was used for 49% (41% to 57%) of births. A paediatrician attended all the births, as was normal practice in Koço Gliozheni Hospital.

For caesarean births a prophylactic uterotonic drug was always given and timing was after the birth, either before cord clamping (7/42) or after cord clamping (35/42). Timing of cord clamping for caesarean births was at 11-20 seconds (86%, 36/42), 21-30 seconds (7%, 3/42), 31-40 seconds (5%, 2/42) or 41-50 seconds (2%, 1/42). Controlled cord traction was not used for caesarean births, all of which had manual removal of placenta.

For preterm births gestation ranged from 31 to 37 weeks. Timing of cord clamping for preterm births was at 11-20 seconds (63%, 10/16), 21-30 seconds (25%, 4/16), 31-40 seconds (6%, 1/16) or 41-50 seconds (6%, 1/16).

## Discussion

Most obstetricians at Koço Gliozheni Hospital reported that they always or usually used active management for the third stage of labour. For timing and choice of the uterotonic drug, reported practice was similar to actual practice observed during the audit. Although some obstetricians reported that they waited longer than one minute before clamping the cord, this was not observed in practice. Controlled cord traction was used for only half the births, and never at caesarean section.

In Albania, ergometrine is not available and so Methergine (methylergonovine maleate), a semi-synthetic ergot alkaloid, is used instead. Other aspects of care during the third stage of labour were similar to those reported from other countries [15,16]. Having a paediatrician present at all births is not usual in other countries; more typical of hospital practice elsewhere would be having a paediatrician present for a fifth of all births [14].

Although our study was conducted at a single hospital in Albania, the results are likely to be representative of other hospitals throughout the country. Koço Gliozheni Hospital is a large tertiary referral centre in the capital city, and a teaching hospital with both medical students and a postgraduate training programme. It therefore has considerable influence on national practice. Usual practice in Albania is that hospital births are attended by an obstetrician, who largely determines care during the third stage. Midwives supervise vaginal births at village health centres, and it is estimated that these total 10% of births in Albania. Home births are rare (less than 1% of total births).

Placental transfusion may increase the infant's circulating blood volume at birth by 20%. For a term infant this equates to an additional 80-100 ml blood,[17,18] which provides 30-40 mg/kg iron,[18,19] sufficient for three months supply for a six month old infant. The volume and duration of placental transfusion may be influenced by factors other than timing of cord clamping, such as whether an uterotonic drug is used before clamping, and gravity due to position of the baby relative to the level of the placenta. For babies born preterm, especially those born before 34 completed weeks, placental transfusion may be particularly important as the expanded blood volume and red cell mass may help the immature infant maintain a stable blood pressure, and improve cardio-respiratory function and organ perfusion.

The evidence from randomised trials is that, for preterm infants, immediate cord clamping increases the need for transfusion in the neonatal period, and the risk of an ultrasound diagnosis of intraventricular haemorrhage, although the effect on grade 3 to 4, the more reliable predictor of long term outcome, is unclear [3,20]. Effects on other outcomes such as jaundice, respiratory distress and hypothermia at birth are less clear, and there are no data on outcome after discharge from hospital. For term babies immediate cord clamping leads to iron deficiency and anaemia in the first few months of life, but with less jaundice and need for phototherapy [4]. Iron deficiency in early childhood is associated with neurodevelopmental delay[21,22], which may be irreversible [23,24]. It is also associated with poor growth, and an increase in recurrent minor infections [25]. Although no follow up studies have reported substantive outcomes, it is plausible that the iron deficiency associated with immediate cord clamping could leave the child more susceptible to infection, and with impaired growth and development in early childhood.

Our data are similar to those from recent surveys in Latin America, Africa and Asia,[16] which suggest that cord clamping within one minute remains widespread clinical practice. This audit of practice in Albania suggests that for over 90% of births the cord is clamped within 30 seconds. Most obstetricians support the need for randomised trials of immediate compared with deferred clamping.

## Conclusions

Most obstetricians reported always or usually using active management for the third stage of labour. For timing and choice of the uterotonic drug, reported practice was similar to actual practice. Although some obstetricians reported they waited longer than one minute before clamping the cord, this was not observed in practice. Controlled cord traction was used for half the births.

## Competing interests

The authors declare that they have no competing interests.

## Contributions of authors

LD conceived the study design. AB and LD prepared the protocol. EN, AD, and AB conducted the study, collected the data, and did the data entry and cleaning. LD did the analysis. LD drafted the paper, with input from AB and EN. All authors read and approved the final manuscript

## Pre-publication history

The pre-publication history for this paper can be accessed here:

http://www.biomedcentral.com/1471-2393/10/4/prepub
